# Fine-Mapping and Identification of a Candidate Gene Underlying the *d2* Dwarfing Phenotype in Pearl Millet, *Cenchrus americanus* (L.) Morrone

**DOI:** 10.1534/g3.113.005587

**Published:** 2013-03-01

**Authors:** Rajiv K. Parvathaneni, Vinod Jakkula, Francis K. Padi, Sebastien Faure, Nethra Nagarajappa, Ana C. Pontaroli, Xiaomei Wu, Jeffrey L. Bennetzen, Katrien M. Devos

**Affiliations:** * Institute of Plant Breeding, Genetics and Genomics, University of Georgia, Athens, Georgia 30602; † Department of Crop and Soil Sciences, University of Georgia, Athens, Georgia 30602; ** Department of Plant Biology, University of Georgia, Athens, Georgia 30602; †† Department of Genetics, University of Georgia, Athens, Georgia 30602; ‡ Cocoa Research Institute of Ghana, New Akim Tafo, Eastern Region, Ghana; § Cereals Genetics and Genomics, Biogemma, 63720 Chappes, France

**Keywords:** comparative genomics, fine-mapping, haplotype analysis, P-glycoprotein

## Abstract

Pearl millet is one of the most important subsistence crops grown in India and sub-Saharan Africa. In many cereal crops, reduced height is a key trait for enhancing yield, and dwarf mutants have been extensively used in breeding to reduce yield loss due to lodging under intense management. In pearl millet, the recessive *d2* dwarfing gene has been deployed widely in commercial germplasm grown in India, the United States, and Australia. Despite its importance, very little research has gone into determining the identity of the *d2* gene. We used comparative information, genetic mapping in two F_2_ populations representing a total of some 1500 progeny, and haplotype analysis of three tall and three dwarf inbred lines to delineate the *d2* region by two genetic markers that, in sorghum, define a region of 410 kb with 40 annotated genes. One of the sorghum genes annotated within this region is *ABCB1*, which encodes a P-glycoprotein involved in auxin transport. This gene had previously been shown to underlie the economically important *dw3* dwarf mutation in sorghum. The cosegregation of *ABCB1* with the *d2* phenotype, its differential expression in the tall inbred ICMP 451 and the dwarf inbred Tift 23DB, and the similar phenotype of stacked lower internodes in the sorghum *dw3* and pearl millet *d2* mutants suggest that *ABCB1* is a likely candidate for *d2*.

Pearl millet [*Cenchrus americanus* (L.) Morrone, previously *Pennisetum glaucum* (L.) R. Br.] (Chemisquy *et al.* 2010) is an important cereal crop grown on an estimated 27 million hectares in Asia and sub-Saharan Africa (FAOSTAT; faostat.fao.org). Being highly drought tolerant, the crop is well adapted to the arid and semiarid tropical environments in these countries. Pearl millet is also grown as a forage crop in regions of the United States, Australia, and South America. Most commercial pearl millet hybrids, whether grown for grain or forage, carry the recessive height-reducing gene *d2* (*e.g.*, Burton 1980; Hanna *et al.* 1997; Gulia *et al.* 2007; Rai *et al.* 2009). The *d2* gene does not affect the length of the coleoptile and mesocotyl (Soman *et al.* 1989) but reduces overall plant height by some 50% through a shortening of all internodes except the peduncle (Burton and Fortson 1966; Burton *et al.* 1969; Rai and Hanna 1990a). Dwarfs tend to yield less grain than their tall isolines, but this negative effect can be mitigated by manipulating the genetic background (Bidinger and Raju 1990; Rai and Rao 1991). Forage quantity is also reduced, but forage quality is greater in the dwarfs than in the talls due to a greater leaf-to-stem ratio (Johnson *et al.* 1968). The greater digestibility of the leaves compared with the stems results in greater animal yields in feed trials (Burton *et al.* 1969; Hanna *et al.* 1979).

The precise origin of the *d2* dwarf mutation is unknown. In the United States, Burton and colleagues discovered in 1939 an extremely leafy pearl millet plant with short internodes among the progeny of a plant obtained through mass selection from five introductions of pearl millet acquired a few years earlier from the Vavilov Institute of Plant Industry, Russia (Burton and Devane 1951; Hein 1953). Based on information in the Germplasm Resource Information Network (*i.e.*, GRIN) database, the five introductions originated from Tunisia (PI 115055), Eritrea (PI 115056, PI 115058), Arabia (PI 115057), and India (PI 115059). The dwarf line was true-breeding and used in crosses with an adapted pearl millet line to form the highly successful synthetic variety ‘Starr’ (Hein 1953). Although there are no records confirming that Starr millet carried the *d2* gene, the described morphology makes this a plausible hypothesis (Kumar and Andrews 1993). Around the same time, in India, Kadam *et al.* (1940) obtained dwarf phenotypes after inbreeding local pearl millet lines. The dwarfs had shortened internodes, overlapping leaf sheaths, and shortened peduncles and were attributed to a recessive mutation. Again, it is unclear whether any of these represented *d2*. In 1966, Burton and Fortson (1966) reported identification of five nonallelic dwarf mutants (*D1*−*D5*). Two of those, *D1* and *D2*, were shown to be controlled by different single recessive genes and were assigned the gene symbols *d1* and *d2*, respectively. The *d2* gene was subsequently incorporated into Indian cultivars through backcross breeding using seed stocks provided by Dr. G. W. Burton (Bakshi *et al.* 1966) and is now widely used in commercial hybrids in the United States, India, and Australia (reviewed by Kumar and Andrews 1993; Gulia *et al.* 2007; Rai *et al.* 2009). The *d2* gene has been mapped on pearl millet linkage group 4 to a 23.2-cM interval flanked by RFLP markers PSM84 and PSM413.2 (Azhaguvel *et al.* 2003).

Height-reducing genes have played key roles in enhancing yield in a range of cereals. The best-known examples are the gibberellic acid (GA)-insensitive *Rht-1* and GA-sensitive *sd-1* dwarfing genes that were essential to the Green Revolutions in wheat and rice, respectively (Peng *et al.* 1999; Monna *et al.* 2002; Sasaki *et al.* 2002), but height mutants have also been widely used in other cereals. For example, in barley, the GA-sensitive *sdw/denso* gene located on chromosome 3H (Laurie *et al.* 1993) has been used extensively in feed and malt cultivars in the Western United States, Canada, Europe, and Australia (reviewed by Mickelson and Rasmusson 1994). Most commercial sorghum lines are “3-dwarf,” which indicates that they carry mutations in three of the four dwarfing genes that have been identified in this species (Schertz *et al.* 1974). The height-reducing gene *Ddw1* on rye chromosome 5R has been deployed in many Eastern-European and Finnish rye breeding programs to develop short-straw cultivars (Milach and Federizzi 2001). A number of these dwarfing genes have been isolated and characterized. The *Rht-1* genes encode DELLA proteins that act as repressors of plant growth (Peng *et al.* 1999). Mutations in the DELLA domain inhibit GA-induced degradation of the DELLA proteins, which results in a GA-insensitive dwarfing phenotype (Peng *et al.* 1997; Dill *et al.* 2001; reviewed by Hedden 2003). The rice *sd-1* gene is a GA 20-oxidase, which catalyzes multiple steps in the GA biosynthetic pathway (Monna *et al.* 2002; Sasaki *et al.* 2002; Spielmeyer *et al.* 2002), and it has recently been shown that the *sdw/denso* gene in barley is likely an ortholog of *sd-1* in rice (Jia *et al.* 2009, 2011). The *dw3* dwarf phenotype in sorghum is caused by an 882-bp tandem duplication in the fifth exon of the *ABCB1* gene. This rearrangement results in the loss of the encoded P-glycoprotein, which modulates polar auxin transport in the stalk (Multani *et al.* 2003).

The aim of our research was to fine-map the *d2* gene in pearl millet and to use comparative information to identify putative candidate genes for the locus. Genetic mapping of an identified candidate gene and preliminary expression analysis provide support for a model that the pearl millet *d2* gene is the ortholog of sorghum *dw3*.

## Materials and Methods

### Mapping populations

**Figure 1:**
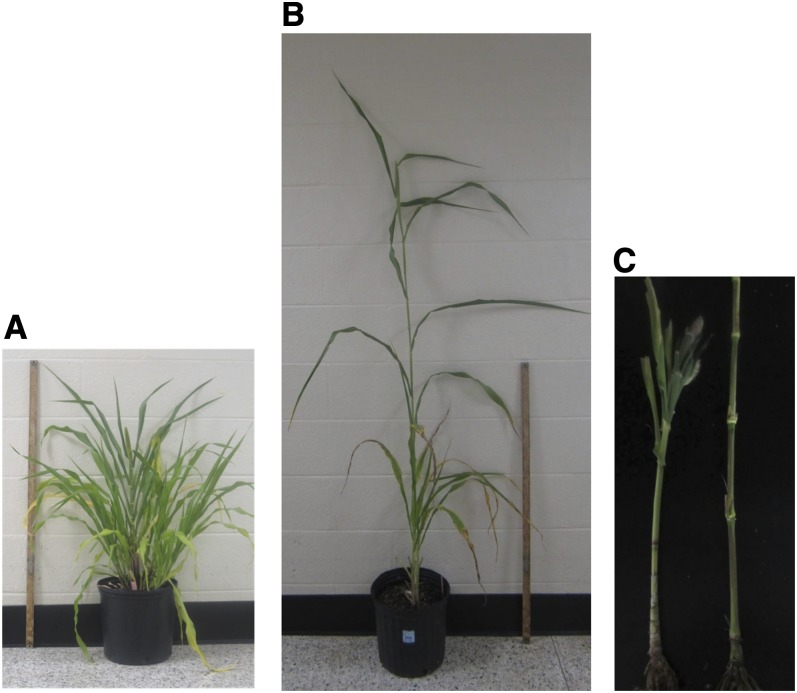
Architecture of (A) inbred Tift 23DB (*d2d2*) and (B) inbred ICMP 451 (*D2D2*), the parents of the fine-mapping population at flowering time (panicle on main tiller 50% exerted). A 1-m ruler is shown for height comparison. Tift 23DB is ~50% shorter and has a greater leaf–to-stem ratio compared with ICMP 451. (C) Phenotype of the stem of Tift 23DB (left) and ICMP 451 (right) after the leaves were removed from the plants shown in (A) and (B) showing stacking of, in particular, the lower internodes in Tift 23DB compared to ICMP 451.

An F_2_ mapping population of a few thousand seed was generated by selfing a single F_1_ hybrid from a cross between the *d2* dwarf inbred Tift 23DB (female; Figure 1A) and the tall inbred ICMP 451 (male; Figure 1B). Tift 23DB was obtained from Wayne Hanna, University of Georgia, Tifton, GA. ICMP 451 was obtained from the International Crops Research Institute for the Semi-Arid Tropics (ICRISAT), Patancheru, India.

A second pearl millet mapping population, originally developed to segregate for a downy mildew resistance gene on linkage group 4, also segregated for *d2*. To construct this population, an F_2_ individual was identified from among the progeny of a genotyped F_2_ population derived from the cross PT 732B (*d2d2*) × P1449-2 (*D2D2*) (Qi *et al.* 2004) that was heterozygous at most marker loci on linkage group 4, including the loci that spanned the region carrying both the resistance gene and the *d2* gene. Twenty-two F_2:3_ plants were grown and analyzed with markers for the region of interest on linkage group 4, and a heterozygous F_3_ plant was selfed to produce a 552 progeny population. In a genetic context, this population, at least in the *d2* region, behaves as an F_2_ population and will be referred to as such. This population was phenotyped for *d2* and mapped with restriction fragment length polymorphism (RFLP) markers in early 2000 (Padi 2002). The Padi (2002) study located the *d2* gene to a 2.8-cM interval between marker PSMP344 and the cosegregating markers B224C4P2 and RGR1963. Because *d2* was scored as a dominant trait (dwarf, *d2d2* and tall, *D2D2* or *D2d2*), the precise position of *d2* could not be determined. However, of the 19 recombination events that could be allocated, 18 events occurred between PSMP344 and *d2*, and 1 occurred between B224C4P2/RGR1963 and *d2*, indicating a tight linkage of *d2* with B224C4P2/RGR1963.

### Bacterial artificial chromosome (BAC) sequencing and sequence analysis

Rice RFLP marker RGR1963 was used to screen a pearl millet BAC library (Allouis *et al.* 2001). Eleven positive clones were identified, of which BAC 293B22 was selected for sequencing. BAC DNA was isolated from clone 293B22 and shotgun libraries prepared as described (Dubcovsky *et al.* 2001). A total of 1152 subclones were sequenced from both ends using Sanger technology. PHRED, PHRAP, and CONSED were used with default parameters for base calling and quality control, sequence assembly, and contig ordering, respectively. The sequence of BAC 293B22 has been deposited in GenBank under accession number KC463796. Gene prediction was performed using FGENESH with the monocot training set (www.softberry.com). Repetitive DNA was identified by BLASTN searches against the Gramineae repeat database (http://plantrepeats.plantbiology.msu.edu/search.html).

### Markers

B224C4P2 is a polymerase chain reaction (PCR)-based marker derived from an end-sequence of pearl millet BAC 224C4, which was one of the 11 clones identified after screening a pearl millet BAC library with the rice RFLP marker RGR1963 (Padi 2002). PSMP344 and PSMP305 are sequence-tagged-site markers derived from RFLP probes PSM305 and PSM344, respectively (Money *et al.* 1994). Three PCR-based markers, Ca_Sb07g023840, Ca_Sb07g023850, and Sb07g023860, were derived from genes identified on BAC 293B22. The prefix Ca stands for *Cenchrus americanus* and is followed by the designation of the sorghum ortholog. In addition, primer sets were developed against 40 genes located in the regions 28.17–28.44 Mb (chromosome end) on rice chromosome 8 and 58.37–59.04 Mb on sorghum chromosome 7. Based on grass comparative data, these rice and sorghum chromosome segments were expected to be syntenic to the pearl millet *d2* region (Devos *et al.* 2000; Devos 2005). Genes selected from the rice and sorghum genomic sequences were used in BLASTN searches to find corresponding expressed sequence tags from other grass species. The genomic and expressed sequence tag sequences were aligned using the program Multalin (Corpet 1988), and primer sets were designed against conserved exon regions flanking an intron. For polymorphism screening and mapping, amplification products were separated on mutation detection enhancement (MDE) acrylamide gels to display single-strand conformation polymorphisms (Martins-Lopes *et al.* 2001). The primer sequences, annealing temperature and location in the sorghum, rice, and *Setaria italica* genomes for all markers are given in Supporting Information, Table S1. To facilitate interpretation of the data, all markers, irrespective of whether they were developed from rice or sorghum, were named after their sorghum ortholog.

### Genotyping

A total of 915 F_2_ individuals from the cross Tift 23DB × ICMP 451 was grown in the glasshouse in batches of 100−200 plants. Genomic DNA was extracted from F_2_ individuals using an ultraquick DNA extraction protocol (Steiner *et al.* 1995) and genotyped with markers B224C4P2 and PSMP305 on MDE gels to identify recombinants in the *d2* region. Plants carrying a recombination event in the *d2* region are referred to as “informative plants.” Informative plants were selfed to produce F_3_ seed. High-quality DNA for further genotyping of the informative plants was obtained either from leaves of the F_2_ plants using a standard CTAB protocol (Murray and Thompson 1980) or from 25 bulked F_3_ seeds using the protocol described by Busso *et al.* (2000). All genotyping was done on MDE gels. DNA fragments were visualized by silver staining (Beidler *et al.* 1982).

PCR amplifications were performed in 20-µL reaction volumes containing 1× PCR buffer (Promega), 1.5 mM MgCl_2_, 0.25 mM of each dNTP, 0.5 µM of forward and reverse primers, 100 ng of template DNA, and 0.8 U of Taq DNA polymerase (Promega). Amplification conditions consisted of an initial denaturation at 94° for 3 min, 38 cycles of denaturation at 94° for 30 sec, primer annealing for 30 sec (see Table S1 for primer-specific annealing temperatures) and extension at 72° for 1 min followed by a final extension at 72° for 5 min. For touch-down PCR (primers indicated with a temperature range in Table S1), the annealing temperature was decreased by 1° every two cycles until the target temperature was reached and 35 PCR cycles were done at the target temperature.

### Phenotyping

To determine the allelic composition at the *d2* locus, 13−25 F_3_ plants were analyzed for each of the informative F_2_ plants. Because of space limitations in the glasshouse, phenotyping was done in multiple batches. Plant height was measured at maturity from the basal node to the top of the panicle (Table S2). Families with a median height <90 cm consisted of plants with the dwarf phenotype, and the corresponding F_2_ genotype was scored as homozygous dwarf (Table 1). Because of the broad range of heights observed for the tall plants, which was caused both by the segregation of other height-affecting genes in the population and environmental effects, we were very conservative in converting the phenotypic measurements to genotypic scores for the *d2* locus. Families with a median height >135 cm were considered to be derived from an F_2_ plant heterozygous at the *d2* locus if the number of plants shorter than 110 cm was not significantly different from 25%. The height of 110 cm was chosen as threshold because less than 2% of dwarf plants were ≥110 cm. F_2_ genotypes giving rise to F_3_ families with a median height between 90 and 130 cm were considered to be either heterozygous or homozygous for the tall allele and were not further classified (Table 1).

**Table 1:** Median height, mean height, and SD of F_3_ families derived from informative F_2_ plants, the number of F_3_ plants per family with height <110 cm, and F_2_ genotypic score

F_2_ plant ID	No. of F_3_ progeny (batch)*^a^*	Median plant height, cm	Mean plant height, cm	SD, cm	Number (percentage) of plants <110 cm*^b^*	F_2_ genotype at *d2* locus
1	23 (1)	85	87	13.99	23 (100%)	*d2d2*
55	23 (1)	139	137.6	22.63	3 (13%)	*D2d2*
177	23 (1)	62	63	11.82	23 (100%)	*d2d2*
263	25 (1)	104	109.1	16.94	ND*^c^*	*D2D2 or D2d2*
310	25 (1)	160	155.4	22.66	0 (0%)	*D2D2*
320	24 (1)	150.5	150.5	21.57	0 (0%)	*D2D2*
344	23 (1)	85	86	17.20	21 (91.3%)	*d2d2*
349	—	—	—	—	—	—
374	23 (2)	89	82.3	18.16		*d2d2*
477	25 (1)	61	64.8	16.04	25 (100%)	*d2d2*
479	22 (1)	155	153	29.27	1 (4.6%)*	*D2D2*
486	24 (1)	107	109.6	30.09	ND	*D2D2 or D2d2*
486	15 (3)	130	121.8	23.21	ND	*D2D2 or D2d2*
496	23 (1)	142	132.5	28.17	4 (17.4%)	*D2d2*
514	21 (2)	121	129.9	34.66	ND	*D2D2 or D2d2*
612	23 (2)	154	150.3	24.35	2 (8.7%)	*D2d2*
701	15 (2)	181	179.8	23.63	0 (0%)	*D2D2*
778	21 (2)	145	145.1	26.81	1 (4.8%)*	*D2d2*
787	17 (3)	157	150.7	26.51	2 (11.8%)	*D2d2*
812	17 (2)	210	203.2	22.54	0 (0%)	*D2D2*
900	13 (2)	189	182	23.88	0 (0%)	*D2D2*
914	14 (2)	192.5	185.9	47.83	2 (14.3%)	*D2d2*
924	22 (2)	186	186.4	24.49	0 (0%)	*D2D2*
930	18 (2)	206	208.4	28.34	0 (0%)	*D2D2*

a F_3_ families with the same batch number were grown concurrently.

b Except where the percentage of plants <110 cm is 0% or 100%, SD from 25% at P ≤ 0.05 is indicated with an asterisk (*).

c Not determined in F_3_ families with a median height between 90 and 135 cm.

### Comparative analyses

The protein corresponding to the primary transcript and its location in the genome was retrieved for all annotated genes in the region between 54.31 and 64.31 Mb on sorghum chromosome 7 (JGI v.1.0) (Paterson *et al.* 2009). Similarly, we retrieved the protein corresponding to the primary transcript for all annotated genes in *S. italica* (35,158 loci in nine chromosomes, JGI v2.1) (Bennetzen *et al.* 2012), *Oryza sativa* (34,781 representative gene loci in 12 chromosomes, IRGSP build 5) (Matsumoto *et al.* 2005), and *Brachypodium distachyon* (23,558 loci in five chromosomes, JGI v.1.0) (Vogel *et al.* 2010). In the first instance, a BLASTP search was carried out with the sorghum proteins as queries against the *S. italica* proteins. The top hit was recorded if the E-value was less than 1e^−5^ and the maximum number of hits at the threshold value was four. Homologous pairs were used to detect syntenic blocks by MCscan (multiple collinearity scan) (Tang *et al.* 2008) and colinear segments were identified using the empirical scoring scheme *min* {−log_10_*E*, 50} for one gene pair and −1 gap penalty for each 10−kb distance between any two consecutive gene pairs. Syntenic blocks with scores >300 and an E-value <1e^−10^ were retrieved. *S. italica* proteins located within these syntenic blocks were then used to identify the syntenic regions in rice and *B. distachyon*. The positions of orthologous gene pairs were plotted using a script in R (R Development Core Team 2005), and the dot plots were used to identify rearrangements. The precise breakpoints of the rearrangements were determined manually.

### Expression analyses

The mapping parents ICMP 451 and Tift 23DB were grown under greenhouse conditions for 7 to 9 wk. The top-most internode and its corresponding nodal leaf were harvested when 50% of the panicle had emerged from the flag leaf sheath. The harvested material was immediately frozen in liquid nitrogen and, if needed, stored at −80°. Total RNA was extracted from leaves and internodes with TRIzol reagent (Chomczynski and Sacchi 1987) and approximately 5−10 µg of isolated RNA was treated with DNase using the Ambion TURBO DNA-*free* kit. cDNA synthesis was performed on ~1 µg of DNase treated RNA using the Roche Transcriptor First Strand cDNA Synthesis kit. The manufacturer’s protocol was used for all experimental steps involving kits. PCR conditions for semi-quantitative PCR consisted of an initial denaturation at 95° for 3 min followed by 29 cycles of denaturation at 95° for 30 sec, annealing at 59° for 30 sec, extension at 72° for 1 min, and a final extension at 72° for 3 min. Primers Ca_Sb07g023730F10 (5′-GCAGGTTCTCCTTGATGCTC-3′) and Ca_Sb07g07g23730R10 (5′-CTCGGAGGCACCTACTTCAC-3′), designed against gene Sb07g023730 (*dw3*), were used to study expression of the pearl millet *dw3* ortholog, whereas actin primers ActinF (5′-ACCGAAGCCCCTCTTAACCC-3′) and ActinR (5′-GTATGGCTGACACCATCACC-3′) were used as internal controls (Van den Berg *et al.* 2004). The expression analysis was conducted twice with samples collected from plants grown at different times of the year.

## Results

### Identifying recombinants in the *d2* region

Mapping of plant height in the PT 732B × P1449-2 population had shown the *d2* gene to be located between the markers B224C4P2 and PSMP344 (Padi 2002). The sequence-tagged-site marker PSMP344 (primer set PSMP344F/R) derived from RFLP marker PSM344 did not amplify from ICMP 451 and thus could only be scored as a dominant marker in the Tift 23DB × ICMP 451 population. Hence, PSMP305, which cosegregates with PSMP344 in most pearl millet maps (Qi *et al.* 2004), was used in combination with B224C4P2 to identify F_2_ plants that carried a recombination event in the *d2* region. Genotyping of 915 F_2_ plants from the cross Tift 23DB × ICMP 451 yielded 29 recombinants, providing an estimate of 1.6 cM for the genetic distance between B224C4P2 and PSMP305. Five plants did not survive the seedling stage, and one plant was removed because of the presence of nonparental alleles, so the fine-mapping and phenotyping was performed on 23 recombinants.

### Determining the genotype at the *d2* locus

The height of the F_2:3_ plants varied both within and between F_3_ families and ranged from 37 to 270 cm (Table S2). Five F_3_ families comprised only dwarf plants, and the corresponding F_2_ plants were genotyped as *d2d2* (Table 1). Five F_3_ families were segregating for plant height in a 3:1 (tall:dwarf) ratio, and the corresponding F_2_ plants were genotyped as *D2d2*. Nine F_3_ families contained either no plants <110 cm (7 families) or a single plant <110 cm (2 families) and the corresponding F_2_ plants were genotyped as *D2D2*. Integrating these data with the genotypic data for markers B224C4P2 and PSMP305 confirmed the location of *d2* in the B224C4P2–PSMP305 interval.

### Marker development and fine-mapping

**Figure 2:**
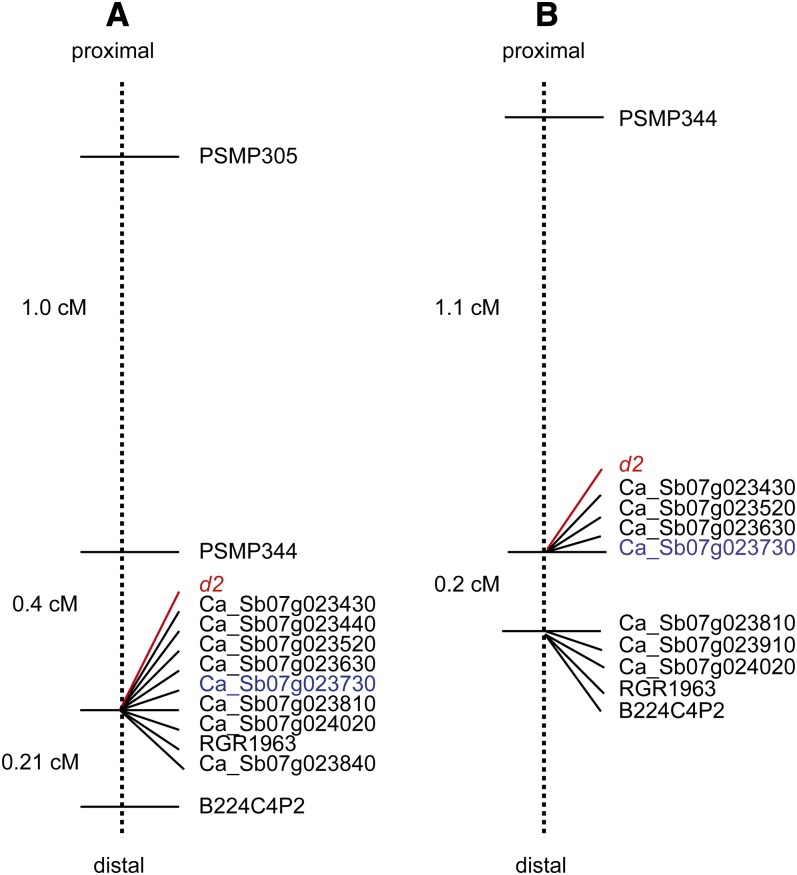
Genetic map of the *d2* region on linkage group 4 of pearl millet generated in (A) the Tift 23DB × ICMP 451 mapping population and (B) the PT 732B × P1449-2 mapping population. The map position of the *d2* phenotype is indicated in red. The map position of the sorghum *dw3* ortholog (Ca_Sb07g023730) is indicated in blue.

Marker RGR1963, which corresponds to sorghum gene Sb07g023850, the two additional markers developed from BAC clone 293B22 that was identified with RGR1963, and the primers designed against selected genes on rice chromosome 8 and sorghum chromosome 7 were tested on Tift 23DB, ICMP 451, and 4 recombinant progeny for their ability to amplify and to detect variation. This initial screen also allowed us to identify markers that would likely map to the *d2* region based on their segregation pattern in the four recombinant lines. Seventy-five percent of the primer sets amplified well in pearl millet, and 30% were polymorphic in the mapping population. Four markers, Ca_Sb07g023460, Ca_Sb07g023470, Ca_Sb07g023500, and Ca_Sb07g023740 had segregation patterns in the four recombinant progeny that were inconsistent with their location in the *d2* region and were not further analyzed. Eight markers corresponding to sorghum genes Sb07g023430, Sb07g023440, Sb07g023520, Sb07g023630, Sb07g023810, Sb07g023840, Sb07g023850, and Sb07g023910 were mapped in the full set of 23 informative progeny. All markers cosegregated (Figure 2A, Table S3). Following the development of a new primer set for PSMP344 (PSMP344F2/R2; Table S1) which amplified in both ICMP 451 and Tift 23DB, PSM344 was mapped between PSMP305 and the cluster of cosegregating markers. Placement of *d2* relative to the fine-mapped markers showed, with the exception of one double recombination event in the *d2* score, complete cosegregation of the *d2* phenotype with the 8-marker cluster (Table S3). The F_3_ family derived from the F_2_ line showing the double recombination event (F_2_ plant with ID 612, Table 1) consisted of 23 plants, 2 of which were 2 and 4 cm shorter than the threshold of 110 cm. Furthermore, although 21:2 did not deviate significantly from a 3:1 ratio, the *P*-value was close to the 5% significance threshold (*P* = 0.071). It seems therefore likely that the genotype for this plant was *D2D2* rather than *D2d2*. When this hypothesis was taken into consideration, we found that the *d2* phenotype cosegregated with the eight-marker cluster (Figure 2A, Table S3).

In an attempt to order the newly generated markers in the pearl millet genome, we selected 16 of the 29 individuals from the PT 732B × P1449-2 population with a recombination event in the interval B224C4P2–PSMP344 for which F_3_ seed was available. Bulked F_3_ seed was grown and seedlings were used for DNA extraction. Mapping of RGR1963, Ca_Sb07g023430, Ca_Sb07g023520, Ca_Sb07g023630, Ca_Sb07g023810, and Ca_Sb07g023910 and one additional sorghum-derived marker, Ca_Sb07g024020, in the 16 informative plants identified two marker clusters (Figure 2B, Table S4). One cluster cosegregated with B224C4P2 and comprised the markers RGR1963, Ca_Sb07g023910, Ca_Sb07g024020, and Ca_Sb07g023810 which, in sorghum, are located in the interval 58.78–59.04 Mb on chromosome 7. The second cluster comprised *d2*, and markers Ca_Sb07g023630, Ca_Sb07g023520, and Ca_Sb07g023430, which were derived from region 58.37–58.54 Mb on sorghum chromosome 7 (Figure 2B, Table S4).

### Comparative analysis of the *d2* region

If we assume that the *d2* region is completely colinear in the pearl millet and sorghum genomes, the ortholog of *d2* should be present in the sorghum genome proximal to location 58.78 Mb (distal boundary). However, our mapping data did not allow us to determine the proximal boundary of the *d2* region. A number of RFLP probes, including PSM344 and PSM305, had previously been end-sequenced (Money *et al.* 1994). The two end-sequences of PSM344, which is a 2-kb probe, mapped 24.7 kb apart on sorghum chromosome 7 (locations 12.59 Mb and 12.61 Mb). For PSM305, one end does not have homology in sorghum at an e-value threshold ≤1e^−05^ whereas the other end identifies sequences on all 10 sorghum chromosomes. We also assessed the location in sorghum of PSM364, which had previously been mapped, depending on the cross, 1.8–6.1 cM distal of PSM305 (Qi *et al.* 2004) but no BLASTN hits were identified. A BLASTN analysis of these same markers in the foxtail millet genome identified hits for the two PSM344 end-sequences 2.7 kb apart at location 9.10 Mb on foxtail millet chromosome VI. One end of PSM305 and both ends of PSM364 identified homologous sequences on foxtail millet chromosome VI at locations 1.36 Mb and 4.18 Mb, respectively. The locations of PSM344, PSM305 and PSM364 in foxtail millet suggest that the region distal to PSM344 is rearranged in pearl millet compared to foxtail millet.

**Figure 3:**
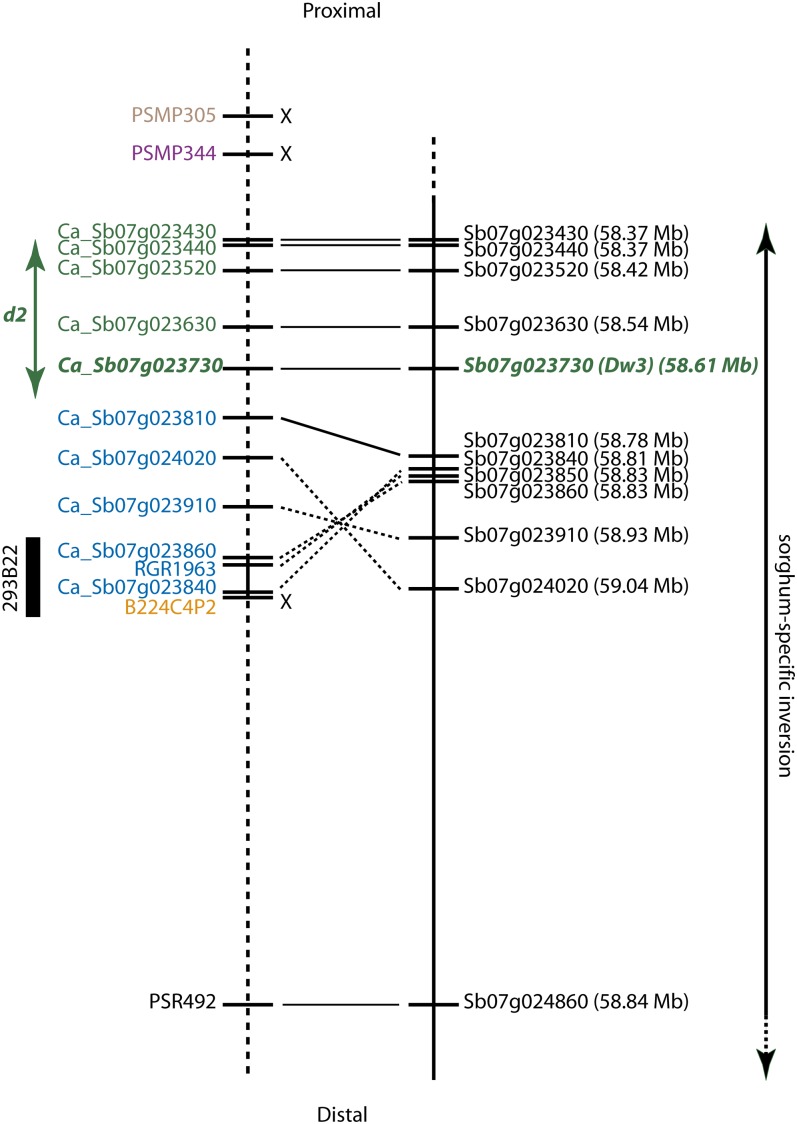
Comparative relationship between the *d2* region in pearl millet (left) and the orthologous region in sorghum (right). Orthologous markers in pearl millet and sorghum are connected with solid lines or, for markers that are inverted in pearl millet relative to sorghum, with dotted lines. Pearl millet markers for which no ortholog could be identified in the depicted sorghum region are indicated with X. Ca_Sb07g023860, RGR1963, Ca_Sb07g023840, and B224C4P2 were located on pearl millet BAC clone 293B22 and distances between those markers are drawn to scale. Distances between other markers in pearl millet are taken from sorghum. Markers shown in the *d2* region in pearl millet in the same color could not be separated by recombination events based on data from both the Tift 23DB × ICMP 451 and PT 732B × P1449-2 mapping populations. Marker Ca_Sb07g023730 (indicated in bold italic) represents the gene underlying the *dw3* phenotype in sorghum. The genome location in sorghum is given in parentheses after the marker name.

Lack of recombination in the pearl millet *d2* region precluded precise ordering of the developed markers. However, three of the mapped markers, Ca_Sb07023860, Ca_Sb07g023840, and RGR1963, were derived from/present on BAC 293B22, which was sequenced to a depth of approximately 12X. The sequence of BAC 293B22 assembled into a single scaffold consisting of three contigs. *De novo* gene identification as well as homology-based annotation identified three genes in the order Ca_Sb07g023860 − 1.7 kb − Ca_Sb07g023850 (which corresponds to RGR1963) − 60.1 kb − Ca_Sb07g023840. Marker B224C4P2 was located 3.1 kb from Ca_Sb07g023840, and both markers were separated by a minimum of one and a maximum of three recombination events in the Tift 23DB × ICMP 451 map (Ca_Sb07g023840 was scored as a dominant marker; hence, not all recombination events could be identified). Combining the genetic mapping data with the gene order information from BAC 293B22 indicated that part of the *d2* region was inverted in pearl millet compared to sorghum (Figure 3).

**Figure 4:**
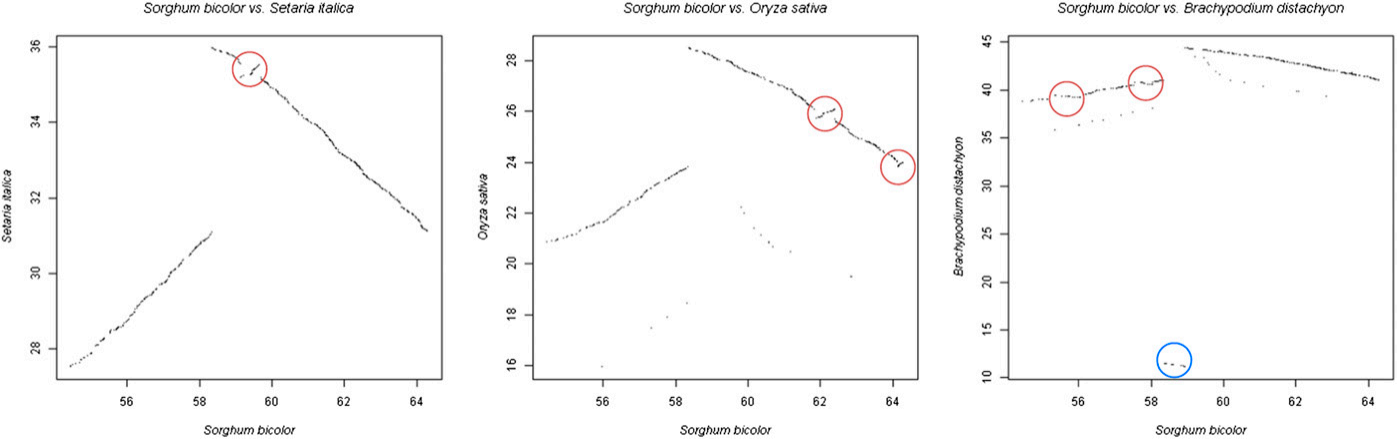
Dot plots showing comparisons at the genome level between region 54.31−64.31 Mb in sorghum and the orthologous regions in *Setaria italica*, *Oryza sativa*, and *Brachypodium distachyon*. Inversions in *S. italica*, *O. sativa* and *B. distachyon* relative to sorghum are circled in red and other rearrangements are circled in blue. Comparison of the three dot plots also shows that the region 58.36–64.31 Mb in sorghum is inverted relative to the other three species.

To better understand the evolution of the *d2* region in grasses, we conducted a comparative analysis at the genome level of the distal 10 Mb of sorghum chromosome 7 (54.31–64.31 Mb). This region was largely colinear between sorghum chromosome 7, foxtail millet chromosome VI, rice chromosome 8, and *B. distachyon* chromosome 3, but a number of species-specific inversions were observed. The distal region of sorghum chromosome 7 (from 58.36 Mb–end) is inverted relative to the other three species (Figure 4, Table S5). This places Si012129m, the foxtail millet ortholog of marker Ca_Sb07g023430, as the most distal marker on foxtail millet chromosome VI for which an ortholog is present on sorghum chromosome 7. In rice, the ortholog of Ca_Sb07g023430 is present on rice chromosome 12 and the rice ortholog to Si015189m, the proximal neighbor of Si013129m, is the last marker on rice chromosome 8 with an ortholog on sorghum chromosome 7. In *B. distachyon*, the ortholog of Ca_Sb07g023430 marks the breakpoint of an ancestral chromosome fusion event. Other rearrangements include an inversion of the region 35.19–35.50 Mb in foxtail millet, two inversions comprising the regions 23.82–23.97 Mb and 25.72–26.07 Mb in rice, and two inversions spanning the regions 40.52–40.80 Mb and 41.65–41.74 Mb in *B. distachyon*. None of these inversions correspond to the inversion that differentiates pearl millet from sorghum.

### Haplotype of the *d2* region in three tall and three dwarf lines

We used the mapped markers to determine the allelic configuration at each of the loci in three dwarf lines, Tift 23DB, PT 732B, and 81B, and three tall lines, ICMP 451, P1449-2, and Tift red (Table 2). At all loci distal to Ca_Sb07g023430, the three dwarf lines carried the same allele, referred to as “a,” whereas the tall lines carried alleles that were different (either “b” or “c”; Table 2) than those observed in the dwarfs. The only exception was at locus Ca_Sb07g023910, where the tall Tift red appeared to have the same allele as the dwarfs. The differentiation between dwarf and tall haplotypes was lost at the three most proximal markers analyzed, Ca_Sb07g023430, PSMP344, and PSMP305.

**Table 2:** Allele composition at 12 loci in three tall and three dwarf inbred lines

	Tall Inbreds			Dwarf Inbreds		
Marker	ICMP 451	P1449-2	Tift red	Tift 23DB	81B	PT 732B
B224C4P2	b	b	b	a	a	a
Ca_Sb07g023840	b	b	b	a	a	a
RGR1963	b	b	b	a	a	a
Ca_Sb07g023910	b	b	a	a	a	a
Ca_Sb07g024020	ND	b	b	ND	ND	a
Ca_Sb07g023810	b	c	b	a	a	a
Ca_Sb07g023630	b	c	b	a	a	a
Ca_Sb07g023520	b	b	b	a	a	a
Ca_Sb07g023440	b	b	c	a	a	a
Ca_Sb07g023430	b	b	a	a	a	a
PSMP344	b	b	a	a	b	a
PSMP305	b	b	a	a	b	a

ND, no data.

### Use of comparative information to identify a putative candidate gene for *d2*

Combining the mapping information with the haplotype data yielded Ca_Sb07g023810 and Ca_Sb07g023430 as the distal and proximal boundary, respectively, of the *d2* region. These two markers defined a 410-kb region in sorghum in which 40 genes had been annotated (www.phytozome.net; Sbi1.4 gene set). The genes, together with their location and functional annotation, are given in Table S6. Sixty-three percent of the 40 genes had no functional annotation. When we focused on the remaining 15 that had homology to characterized proteins, we found that Sb07g023730 became a likely candidate for *d2* because it had previously been identified as the gene underlying the *dw3* and *br2* dwarf phenotypes in sorghum and maize, respectively (Multani *et al.* 2003). Sb07g023730, an ABC transporter of the B subfamily (member 1), encodes a P-glycoprotein that modulates auxin transport in the stalk (Multani *et al.* 2003; Knoller *et al.* 2010).

### Preliminary validation of *ABCB1* as a candidate for *d2*

Several forward and reverse primers were designed against the sequence of gene Sb07g023730. One primer combination, Ca_Sb07g023730F1/R5 (Table S1), yielded a strong amplification product in the tall inbreds ICMP 451, P1449-2 and Tift red but did not amplify in the dwarf inbreds Tift 23DB, PT 732B and 81B. Sanger sequencing of the fragment amplified from the tall inbred ICMP 451 (File S1) and BLASTX analysis of the resulting sequence to the ‘nr’ section of GenBank confirmed that the amplified fragment was derived from gene *ABCB1* (96% identity with both the sorghum and maize ABCB1 protein). Mapping of this product in the informative progeny of the Tift 23DB × ICMP 451 and PT 732B × P1449-2 F_2_ populations showed Ca_Sb07g023730 to fall within the cluster of markers that cosegregated with *d2* (Figure 2, A and B). This finding demonstrated that the pearl millet ortholog of Sb07g023730 was located within the *d2* interval.

**Figure 5:**
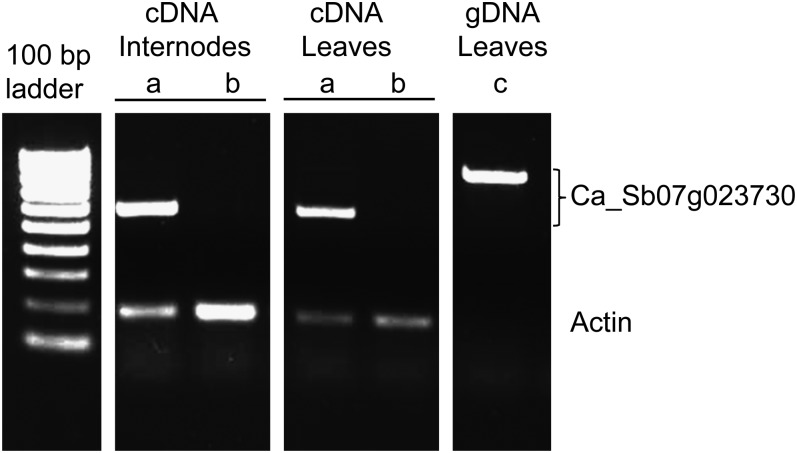
Semiquantitative RT-PCR with primers designed against sorghum gene Sb07g023730 showing a 564-bp fragment in cDNA extracted from leaves and internodes of ICMP 451 (*D2D2*) (lane a) and no/a weak fragment of the same size in cDNA extracted from leaves and internodes of Tift 23DB (*d2d2*) (lane b). Lane c shows the 841-bp fragment obtained using genomic DNA from the tall inbred ICMP 451. Primers homologous to an actin gene were used as an internal control.

A second primer set Ca_Sb07g023730F10/R10 gave strong amplification products in both ICMP 451 and Tift 23DB. Sequence analysis showed that the ICMP 451 and Tift 23DB fragments differed by a single synonymous SNP (File S2). Because this SNP could not be visualized by single-strand conformation polymorphism gel electrophoresis, the amplicon obtained with primer set Ca_Sb07g023730F10/R10 was not mapped. A BLASTX analysis, however, confirmed that the Ca_Sb07g023730F10/R10 amplification product corresponded to *ABCB1* (92% identity with both the sorghum and maize ABCB1 protein). This primer set, which flanked introns 2 and 3 in Sb07g023730, was used in a semiquantitative reverse transcriptase (RT)-PCR experiment and showed that Ca_Sb07g023730 was differentially expressed in both the top internode and corresponding leaf between ICMP 451 (*D2D2*) and Tift 23DB *(d2d2)* (Figure 5). The expression data provided support for our hypothesis that Sb07g023730 is the *d2* gene.

## Discussion

### Organization of the *d2* region

To our knowledge, no traits have been fine-mapped in pearl millet. The *d2* dwarfing gene, despite its widespread incorporation into commercial germplasm, had previously only been located to a 23.2-cM interval on pearl millet linkage group 4 (Azhaguvel *et al.* 2003). We initially mapped *d2* to a 1.6-cM region, but attempts to further narrow the interval in an approximately 1000 progeny F_2_ population largely failed due to a lack of recombination. Markers developed from a 670-kb region on sorghum chromosome 7 (58.37–59.04 Mb) all cosegregated with the *d2* phenotype. Recombination in pearl millet is very unevenly distributed (Liu *et al.* 1994; Qi *et al.* 2004) and it might be that the *d2* region has inherently low recombination rates. An alternative explanation is that the two mapping parents, ICMP 451 and Tift 23DB, differ by an inversion in this region. Combining sequence information from a BAC clone originating from the *d2* region with recombination data on the four markers that were identified on this BAC indicated the presence of an inversion in the *d2* region in Tift 23DB relative to sorghum (Figure 3). Although we could not precisely determine the boundaries of the inversion, the fact that markers Ca_Sb07g023810 and PSR492 (which is orthologous to Sb07g024860) were located outside the inversion suggests that the inversion likely encompasses less than 100 genes. As the pearl millet genome is characterized by a large number of chromosomal rearrangements relative to other grass genomes (Devos *et al.* 2000), it was not particularly surprising to observe this inversion at the *d2* locus. However, it is unclear whether the inversion is present in all pearl millet lines or if it is limited to the *d2* dwarfs or, possibly, even the inbred Tift 23DB. We therefore cannot exclude the possibility that the lack of recombination seen in this region between Tift 23DB and ICMP 451 is the result of the differential presence of this rearrangement in the two parental lines. The pattern of recombination seen in the PT 732B × P1449-2 mapping population was consistent with both overall reduced recombination and the presence of an inversion in one of the parents, and hence did not provide further insights into the specific organization of the *d2* region.

### Comparative analysis of the *d2* region

Comparative information has been used to develop markers for specific chromosome regions and, in some cases, to identify candidate genes underlying traits (Kilian *et al.* 1997; Yan *et al.* 2003, 2004). In pearl millet, comparative relationships are complicated by the extensive chromosomal rearrangements that have taken place in the pearl millet genome since its divergence from a common ancestor with foxtail millet some 8.3 million years ago (Devos *et al.* 2000; Bennetzen *et al.* 2012). Although there are few data on comparative relationships at the DNA sequence level for pearl millet, a comparative study involving *Aegilops tauschii*, rice, sorghum, and *B. distachyon* suggests that the relative frequency with which gross chromosomal rearrangements and small-scale rearrangements, mainly the insertion of duplicated gene copies, occur is significantly correlated (Massa *et al.* 2011). Therefore, disruption of colinearity at the gene level might be greater in pearl millet relative to the other grasses. However, even if the larger number of chromosomal rearrangements in pearl millet relative to other grasses means a greater number of gene insertions, the expectation is still that gene orders will have remained sufficiently conserved to exploit comparative relationships for marker development. Nine of the 13 markers developed from the orthologous sorghum region that were polymorphic in the mapping population mapped to the *d2* region in pearl millet. The other four primer sets generated segregation patterns in the preliminary screen, which consisted of the parents and four recombinant progeny, indicating to us that the polymorphic fragments were located outside the *d2* region. Because we did not attempt by sequence analysis to establish orthology between the scored pearl millet fragments and the sorghum genes used for primer design, we cannot state with certainty that those four genes are located in noncolinear positions in pearl millet and sorghum. Two of those, Sb07g023460 and Sb07g023470, are found in colinear positions in sorghum, foxtail millet, rice, and *B. distachyon*. The other two are either located in noncolinear positions or have duplicated gene copies in noncolinear positions in at least some of the sequenced grass genomes (www.gramene.org).

Although gene orders were overall highly conserved between the regions orthologous to *d2* in foxtail, sorghum, rice, and *B. distachyon*, species-specific inversions were identified in all four species. The entire distal region of sorghum chromosome 7 had undergone an inversion with a breakpoint between 58.34 and 58.36 Mb, which meant that the genes that were located immediately distal to the inversion breakpoint region in sorghum had been located near the telomere in the ancestral grass genome. The ancestral distal position was maintained only in foxtail millet and rice. In sorghum, *B. distachyon*, and pearl millet, chromosomal rearrangements had moved the ancestral telomere to an interstitial position. This almost certainly had been accompanied by a reduction in recombination, in particular in pearl millet, where a very strong recombination gradient exists along the chromosomes from the centromere to the telomere (Qi *et al.* 2004). Although all the inversions observed were species-specific, it is interesting to note that the distal inversion in sorghum and the 23.82–23.97 Mb inversion in rice have one of their breakpoints in common, suggesting that the breakpoint might represent a region on the ancestral grass chromosome that is prone to breakage.

### *ABCB1* as a candidate for *d2*

Our mapping data had indicated that the distal boundary for the *d2* region in sorghum was at location 58.78 Mb on sorghum chromosome 7, but we had not been able to establish a proximal boundary due to cosegregation of the markers with the *d2* phenotype. However, haplotype analysis of three tall and three dwarf inbred lines with the mapped markers suggested that the *d2* gene was located distal to marker Ca_Sb07g023430, whose ortholog is located at position 58.37 Mb in sorghum. In the region spanned by the markers B224C4P2 and Ca_Sb07g023440, the allelic composition of the dwarf inbreds was identical and different from that of the tall inbreds, except at locus Ca_Sb07g023910 in line Tift red (Table 2). This finding suggests that the three dwarfs analyzed (Tift 23DB, PT 732B, and 81B) are derived from the same *d2* source. As expected, we find different haplotypes in this region in the tall inbreds ICMP 454, P1449-2, and Tift red (Table 2). The inbred 81B is a downy mildew resistant selection from gamma-irradiation treated Tift 23DB (Rai and Hanna 1990b). Although this line maintains the dwarf haplotype in the *d2* region, it likely underwent a recombination event with a tall line between markers Sb07g023430 and PSMP344 (Table 2). Tift red is a backcross line produced by the late Glenn Burton that carries a gene for purple plant color and the tall *D2* allele in a Tift 23 background. Considering that no recombination was identified between Ca_Sb07g023430 and Ca_Sb07g023440 in the ~1500 progeny we analyzed from two crosses, it was surprising to see that Tift 23DB and Tift red, which are near-isogenic lines, differ by a recombination event between those two markers. The unexpected haplotype of Tift red was crucial to determining the proximal boundary of the *d2* region.

The region delineated in sorghum as being orthologous to the *d2* region in pearl millet contained the adenosine triphosphate−binding cassette (ABC) subfamily B1 gene, an obvious candidate for *d2* because a mutation in this gene was shown to underlie the recessive sorghum *dw3* dwarfing phenotype (Multani *et al.* 2003). A mutation in the same gene is also responsible for the brachytic2 (*br2*) phenotype in maize. The ABCB1 protein belongs to the multidrug resistant class of P-glycoproteins and plays a role in auxin transport in the nodal/intercalary meristem regions (Knoller *et al.* 2010). Consequently, in the *br2/Zmpgp1* and *dw3/Sbpgp1* mutants, auxin accumulates in the vicinity of the nodes. Because auxin is synthesized mainly in the shoot apex and young leaves, and then transported basipetally, the lowermost internodes in the sorghum *dw3* and maize *br2* mutants are affected the most by the modulation of auxin transport caused by a knockout of the *ABCB1* gene (Multani *et al.* 2003; Knoller *et al.* 2010). The phenotype of stacked lower internodes in the pearl millet *d2* dwarf is very similar to that observed in sorghum *dw3* and maize *br2* mutants (Figure 1C).

In an attempt to validate *ABCB1* as a candidate for *d2*, we designed multiple primer sets against the sorghum *ABCB1* gene and tested them in three dwarf and three tall lines. One primer set, which spanned intron 1, yielded an amplification product in all tall lines tested and in none of the dwarfs, and this polymorphism cosegregated with the height phenotype in the set of informative F_2_ plants in both the Tift 23DB × ICMP 451, and PT 732B × P1449-2 populations. The most likely cause for the lack of amplification in the dwarf lines is either a single-nucleotide polymorphism or deletion at a primer site that prohibits primer extension or the presence of an insertion in the region between the two primer binding sites that extends the fragment to be amplified beyond the length limit of a typical PCR. More work is needed to determine whether the observed variation could be the underlying cause of the dwarf phenotype.

We also analyzed the expression of the *ABCB1* gene in the topmost internode and the corresponding leaf in flowering plants of one *d2* dwarf plant, Tift 23DB, and one tall plant, ICMP 451, using semiquantitative RT-PCR. The RT-PCR yielded a strong amplification product in both tissues in ICMP 451 and a weak product in Tift 23DB, which suggests that *ABCB1* is differentially expressed in the tall and dwarf inbreds in both tissues or that the stability of the *ABCB1* mRNA is reduced in the dwarf mutant (Figure 5). A reduced transcript level in the dwarf mutant is in agreement with the recessive nature of the *d2* mutation. In maize, *ABCB1* is expressed in nodal tissue and possibly in internodes, although reports on the latter are not consistent (Multani *et al.* 2003; Knoller *et al.* 2010). No expression was detected in maize leaves (Multani *et al.* 2003). Nodes were not included in our preliminary expression analysis because of the difficulty of extracting RNA from the hard node tissue. In Arabidopsis, *ABCB1* expression is highest in nodes, but the gene is also expressed in a range of other tissues (Titapiwatanakun and Murphy 2009). More detailed expression analyses are needed in pearl millet to determine precisely where the *ABCB1* gene is expressed and at what levels. The greater expression in the tall compared with the dwarf inbred, however, provided some support for our hypothesis that *ABCB1* is a reasonable candidate for *d2*.

In conclusion, using a combination of genetic mapping, haplotype analysis and comparative genomics, we have fine-mapped the pearl millet *d2* dwarf phenotype to a region which, in sorghum, spans 410 kb and contains 40 annotated genes. A candidate gene, *ABCB1*, was identified as putatively underlying *d2*. Work is currently underway to isolate full-length copies of the *ABCB1* gene from both a tall and a dwarf inbred to further test our candidate gene hypothesis. In the meantime, our work provides breeders with a set of markers that can be used to identify the presence of the recessive *d2* gene in heterozygous condition and at the seedling stage. Phenotypically, the dwarf phenotype can only be scored in homozygous condition and at the booting stage, so the markers will enhance the efficiency of breeding programs that use tall lines as sources of novel genes for the improvement of dwarf inbreds.

## Supplementary Material

Supporting Information
