# Supplementary material for: Fine-Mapping and Identification of a Candidate Gene Underlying the d2 Dwarfing Phenotype in Pearl Millet, Cenchrus americanus (L.) Morrone
Source: G3 (Bethesda). 2013 Mar 1;3(3):563–72. doi: 10.1534/g3.113.005587 (PMC3583462; doi:10.1534/g3.113.005587)
Supplement: Supporting Information [file supp_3_3_563__index.html]

Supporting Information 

# Fine-Mapping and Identification of a Candidate Gene Underlying the *d2* Dwarfing Phenotype in Pearl Millet, *Cenchrus americanus* (L.) Morrone

## Supporting Information for Parvathaneni *et al.*, 2013

**Files in this Data Supplement:**

- Supporting Information - Files S1 and S2 and Tables S1-S6 (PDF, 220 KB)
- File S1 - Sequence of DNA fragment obtained with primer set Ca\_Sb07g023730F1/R5 in the tall inbred line ICMP 451 (PDF, 64 KB)
- File S2 - Sequence of DNA fragments obtained with primer set Ca\_Sb07g023730F10/R10 in the tall inbred line ICMP 451 (A) and in the dwarf inbred line Tift 23DB (B). The synonymous SNP that differentiates the ICMP 451 and Tift 23DB sequences is shown in red (PDF, 68 KB)
- Table S1 - Primer sequences, their melting temperature and location in the sorghum, rice and foxtail millet genomes of markers mapped in the *d2* region (PDF, 96 KB)
- Table S2 - Height of individual plants within F3 families derived from informative F2 plants (.xlsx, 25 KB)
- Table S3 - Genotypic scores for *d2* and 12 markers in 23 informative F2 plants from the cross Tift 23DB x ICMP 451 (.xlsx, 12 KB)
- Table S4 - Genotypic scores for *d2* and 11 markers in 16 informative F2 plants from the cross PT 732B x P1449-2 (.xlsx, 12 KB)
- Table S5 - Annotated genes in the distal 10 Mb of sorghum chromosome 7 and their orthologs in *Setaria italica*, *Oryza sativa* and *Brachypodium distachyon* (.xlsx, 84 KB)
- Table S6 - Genes annotated in the sorghum genome in the region flanked by Sb07g023430 and Sb07g023810 (.xlsx, 11 KB)
