# Supplementary material for: Fine-Mapping and Identification of a Candidate Gene Underlying the d2 Dwarfing Phenotype in Pearl Millet, Cenchrus americanus (L.) Morrone
Source: G3 (Bethesda). 2013 Mar 1;3(3):563–72. doi: 10.1534/g3.113.005587 (PMC3583462; doi:10.1534/g3.113.005587)
Supplement: Supporting Information [file supp_3.3.563_005587SI.pdf]

G3 Genes | Genomes | Genetics

Fine-mapping and identification of a candidate gene underlying the *d2* dwarfing phenotype in pearl millet, *Cenchrus americanus* (L.) Morrone.

Rajiv K. Parvathaneni[*,#], Vinod Jakkula[§,#,1] Francis K. Padi[**], Sebastien Faure[§§], Nethra N. [*,§§§,1], Ana C. Pontaroli[***,1], Xiaomei Wu[*,§§§,1], Jeffrey L. Bennetzen[***] and Katrien M. Devos[*,§,§§§]

[*] Institute of Plant Breeding, Genetics and Genomics, University of Georgia, Athens, GA 30602, USA
[§] Department of Crop and Soil Sciences, University of Georgia, Athens, GA 30602, USA
[**] Cocoa Research Institute of Ghana, P.O. Box 8, New Akim Tafo, Eastern Region, Ghana
[§§]Cereals Genetics and Genomics, Biogemma, Route d'Ennezat, CS90126, 63720 Chappes, France
[***]Department of Genetics, University of Georgia, Athens, GA 30602, USA
[§§§] Department of Plant Biology, University of Georgia, Athens, GA 30602, USA

[1]Current address: **ACP**: EEA Balcarce, Instituto Nacional de Tecnología Agropecuaria (INTA) - Consejo Nacional de Investigaciones Científicas y Técnicas (CONICET), CC 276 (7620) Balcarce, Argentina; **NN:** AICRP on Seed Technology, NSP, GKVK, University of Agricultural Sciences, Bangalore 560 065, India; **VJ:** Monsanto Company, 110 T.W. Alexander Drive, Research Triangle Park, NC 27709; **XW:** Center for Plant Environmental Sensing, and College of Life and Environmental Sciences, Hangzhou Normal University, Hangzhou 310036, China

Corresponding author:

Katrien M. Devos
Dept. of Crop and Soil Sciences
3111 Miller Plant Sciences
University of Georgia
Athens, GA 30602
USA
Phone: 706-542-0925
Fax: 706-542-0914
E-mail: kdevos@uga.edu

DOI: 10.1534/g3.113.005587

File S1

Sequence of DNA fragment obtained with primer set Ca_Sb07g023730F1/R5 in the tall inbred line ICMP 451.

```
ICMP 451_Ca_Sb07g023730F1R5
TACGCCTTCTACTTCCTCGTCGTCGGGGCAGCCATCTGGGCATCCTCCTGGGCCGAGATCTCCTGCTGGAT
GTGGACCGGCGAGCGCCAGTCCACCCGGATGCGCATCCGCTACCTCGACGCCGCCCTCCGCCAGGACGTCT
CCTTCTTCGACACCGACGTCCGCGCCTCCGACGTCATCTACGCCATCAACGCCGACGCCGTCCTCGTCCAG
GACGCCATCAGCGAGAAGCTCGGCAACCTCATCCACTACATGGCCACCTTCGTCGCCGGATTCGTCGTCGG
CTTCACCGCCGCCTGGCAGCTCGCGCTCGTCACGCTCGCCGTCGTACCGCTCATCGCCGTCATCGGGGGGC
TCAGCGCCGCCGCGCTCTCCAAGCTCTCCGCCAGGAGCCAGGACGCGCTCTCCGGCGCCAGCGCCATCGCC
GAGCAGGCGCTGGCGCAGATACGGATCGTGCAGGCCTTCGTCGGCGAGGAGCGCGCCATGCGGGCGTACTC
GGCGGCGCTGGCCGTCGCGCAGAAGATCGGCTACCGGAGCGGCGTCGCCAAGGGGCTCGGGCTCGGCGGCA
CCTACTTCACCGTCTTCTGCTGCT
```

File S2

Sequence of DNA fragments obtained with primer set Ca_Sb07g023730F10/R10 in the tall inbred line ICMP 451 (A) and in the dwarf inbred line Tift 23DB (B). The synonymous SNP that differentiates the ICMP 451 and Tift 23DB sequences is shown in red.

```
A. ICMP 451_Ca_Sb07g023730F10/R10
GCAGGTTCTCCTTGATGCTCGTCGCGAACAGCGTCGGCTCCTGACTCACCAGGCCCATCTGCTGCCGGAGC
CACCGGAGCTTCAGGCTCTTGAGGTCATGCCCGTCCAGCAGGATCTCACCTGTTGCAATTGCAATTCCCAT
CGCTCATCAGAATAGTAGTTTGTTTTCTAGTAGTAGACTTTTCTGCCTATTACTAGAGGATGCCTATGAAT
GACTGTGACTGGACAAGAGGATGATGCAGCGCAAAGGAAGCAGCCCATGAGGCCGGCAAGCTCTGCTCCAA
TCCAAGCACTACTTGCTTGGTTGCTTGCACCACCCCCACCCAGCAATGGCGGGCAATGCTGATTACTGATG
GCTCCAATCCAACAAGAGAATGTAACCAACAACGGGTATACCTGCGCTGGGGTCGTAGAACCTCTCGATGA
GCGACACGACGGTGCTCTTCCCGGAGCCGGAGCTGCCGACCAGCGCGATGGTCTTCCCGGCGGGCACGGTG
AGGGAGAAGGCGCGCAGGATGGGCACGTCCGGCCGCGTCGGGTACGCGAAGTCGACGCCCCGCATCTCTAC
CCGCCCCGTGACGGACTCCAGCTCCACCTGGCCGTCCCGCGAGATCCCCGGCTTGTGGTCGATGATGCGGA
AGATCTTGGCCGCCGCGACGCGCGCCTTGGCGAAGGCGGCCATGCTCGGCGCCGACTGCCCCAGCGCGATG
CCCCCGATCATGACGGAGAACATGGTGGCGATGGCGAGCCCCCCGTTGGTGCGGTGGCCGCGCACCAGGTG
GCCCCCGTACCAGAGCAGCAGCCCGTAGCAGCAGAAGACGGTGAAGTAGGTGCCTCCGAG

B. Tift 23DB_Ca_Sb07g023730F10/R10
GCAGGTTCTCCTTGATGCTCGTCGCGAACAGCGTCGGCTCCTGACTCACCAGGCCCATCTGCTGCCGGAGC
CACCGGAGCTTCAGGCTCTTGAGGTCATGCCCGTCCAGCAGGATCTCACCTGTTGCAATTGCAATTCCCAT
CGCTCATCAGAATAGTAGTTTGTTTTCTAGTAGTAGACTTTTCTGCCTATTACTAGAGGATGCCTATGAAT
GACTGTGACTGGACAAGAGGATGATGCAGCGCAAAGGAAGCAGCCCATGAGGCCGGCAAGCTCTGCTCCAA
TCCAAGCACTACTTGCTTGGTTGCTTGCACCACCCCCACCCAGCAATGGCGGGCAATGCTGATTACTGATG
GCTCCAATCCAACAAGAGAATGTAACCAACAACGGGTATACCTGCGCTGGGGTCGTAGAACCTCTCGATGA
GCGACACGACGGTGCTCTTCCCGGAGCCGGAGCTGCCGACCAGCGCGATGGTCTTCCCGGCGGGCACGGTG
AGGGAGAAGGCGCGCAGGATGGGCACGTCCGGCCGCGTCGGGTACGCGAAGTCGACGCCCCGCATCTCTAC
CCGCCCCGTGACGGACTCCAGCTCCACCTGGCCGTCCCGCGAGATCCCCGGCTTGTGATCGATGATGCGGA
AGATCTTGGCCGCCGCGACGCGCGCCTTGGCGAAGGCGGCCATGCTCGGCGCCGACTGCCCCAGCGCGATG
CCCCCGATCATGACGGAGAACATGGTGGCGATGGCGAGCCCCCCGTTGGTGCGGTGGCCGCGCACCAGGTG
GCCCCCGTACCAGAGCAGCAGCCCGTAGCAGCAGAAGACGGTGAAGTAGGTGCCTCCGAG
```

Table S1 Primer sequences, their melting temperature and location in the sorghum, rice and foxtail millet genomes of markers mapped in the *d2* region

Marker		Primer sequences	Tm (^{0}C)	Corresponding sorghum gene	Location in sorghum genome (Mb)[1]	Corresponding rice gene	Location in rice genome (Mb)[1]	Corresponding foxtail millet gene	Location in foxtail millet genome (Mb)[1]
B224C4P2	F2	TTCCTGGGATCAAAAGGTTC	59-52	-	-	-	-	-	-
	R1	CCATATCTCCAAGCTGCATAG							
Ca_Sb07g024020	F	CCACGCGCTTCTTCCTCC	61	Sb07g024020	7: 59.04	Os08g0560300	8: 28.15	Si015992m	VI: 35.69
	R	TCCTCCACCTCGGACACG							
Ca_Sb07g023910	F	CATGCACTGACGAGGTTGG	53	Sb07g023910	7: 58.93	Os080562100	8: 28.23	Si013632m	VI: 35.76
	R	TGCCCCATTTTTGGATGAGC							
RGR1963	2F	GTACGCCTACAGTCAAGGTG	59-52	Sb07g023850	7: 58.82	Os08g0562700	8: 28.27	Si013315m	VI: 35.80
	2R	CCAAGCTCGTGTTGCGCAG							
Ca_Sb07g023840	F	ACATCCTCCCCTCCGTATTC	53	Sb07g023840	7: 58.81	Os08g0562800	8: 28.27	Si013571m	VI: 35.81
	R	ATGGAGAAGCTGTCCCTGAAG							
Ca_Sb07g023810	F	CTTGGCTCACCATGTGGTG	61	Sb07g023810	7: 58.78	Os08g0563400	8: 28.30	Si015156m	VI: 35.83
	R	GACTCTAGTTCTGCTTGCAG							

Ca_Sb07g023730	F1	TACGCCTTCTACTTCCTCGTC	61	Sb07g023730	7: 58.61	Os080564300	8: 28.36	Si013123m	VI: 35.86
	R5	AGCAGCAGAAGACGGTGAAGTAG							
Ca_Sb07g023630	F	GCTCTTTTCAGCAACCCCTTC	53	Sb07g023630	7: 58.53	Os08g0564800	8: 28.39	Si013125m	VI: 35.88
	R	CGCTAACTGCAGCAGCAAGG							
Ca_Sb07g023520	F	GCCATTTTGGCGGAGATTGC	53	Sb07g023520	7: 58.42	Os080566000	8: 28.44	Si013435m	VI: 35.92
	R	CTGCGCACCAGCAATAGG							
Ca_Sb07g023500	F	GCTTTAGACGGTGCAGAC	59-52	Sb07g023500	7: 58.40	Os08g0566400	8: 28.46	Si015293m	VI: 35.94
	R	GAAGCTCATCCAATCCCTC							
Ca_Sb07g023470	F	AGCTTCAGCTCCGTGCTTC	59-52	Sb07g023470	7: 58.39	Os080566600	8: 28.47	Si015371m	VI: 35.95
	R	GGAATCCGAGCTTCTCTCC							
Ca_Sb07g023460	F2	GCATCTGTCCAACTGACAGC	59-52	Sb07g023460	7: 58.38	Os080566900	8: 28.48	Si014505m	VI: 35.95
	R2	ATCTTGCTGCCTTGAGGTTC							
Ca_Sb07g023440	F	CGCCTTCGACATGGACGAC	53	Sb07g023440	7: 58.37	Os080567100	8: 28.49	Si015189m	VI: 35.96
	R	CTTGTCGTGGCGGTAGTCG							
Ca_Sb07g023430	F	TGATAAACCCAGCAACCTTGG	53	Sb07g023430	7: 58.37	Os12g0641500	12: 27.75	Si013129m	VI: 35.97
	R	GAATCAAGTACACAGCTATGC							

PSMP344	F	CGTCCTGTACCTCAGCCTTG	59-52	Sb07g007800	7: 12.59-12.61	-	-	Si015622	VI: 9.10
	R	ATGCTACACCTGTCAGAACTAAGAG							
PSMP344	F2	GGCTTGTCACGTCCTGTACC	53	Sb07g007800	7: 12.59	-	-	Si015622	VI: 9.10
	R2	TCAATCATGCAGCAGCAATC							
PSMP305	F	TGCAATAGTTTCCAGTTGTC	53	-	-	-	-	-	VI: 1.36
	R	TCAACAATTTGGTACAGACG							

[1]The number before the colon indicates the chromosome number; the number following the colon is the chromosomal location in Mb.

Tables S2-S6

Table S2: Height of individual plants within F_3 families derived from informative F_2 plants.

Table S3: Genotypic scores for *d2* and 12 markers in 23 informative F2 plants from the cross Tift 23DB x ICMP 451

Table S4: Genotypic scores for d2 and 11 markers in 16 informative F2 plants from the cross PT 732B x P1449-2

Table S5: Annotated genes in the distal 10 Mb of sorghum chromosome 7 and their orthologs in *Setaria italica*, *Oryza sativa* and *Brachypodium distachyon*. For each gene, the start and end positions are given.

Table S6: Genes annotated in the sorghum genome in the region flanked by Sb07g023430 and Sb07g023810

Tables S2-S6 are available for download at http://www.g3journal.org/lookup/suppl/doi:10.1534/g3.113.005587/-/DC1.
