# Supplementary material for: Fine-Mapping and Identification of a Candidate Gene Underlying the d2 Dwarfing Phenotype in Pearl Millet, Cenchrus americanus (L.) Morrone
Source: G3 (Bethesda). 2013 Mar 1;3(3):563–72. doi: 10.1534/g3.113.005587 (PMC3583462; doi:10.1534/g3.113.005587)
Supplement: Supporting Information [file supp_3.3.563_FileS1.pdf]

File S1

Sequence of DNA fragment obtained with primer set Ca_Sb07g023730F1/R5 in the tall inbred line ICMP 451.

```
ICMP 451_Ca_Sb07g023730F1R5
TACGCCTTCTACTTCCTCGTCGTCGGGGCAGCCATCTGGGCATCCTCCTGGGCCGAGATCTCCTGCTGGAT
GTGGACCGGCGAGCGCCAGTCCACCCGGATGCGCATCCGCTACCTCGACGCCGCCCTCCGCCAGGACGTCT
CCTTCTTCGACACCGACGTCCGCGCCTCCGACGTCATCTACGCCATCAACGCCGACGCCGTCCTCGTCCAG
GACGCCATCAGCGAGAAGCTCGGCAACCTCATCCACTACATGGCCACCTTCGTCGCCGGATTCGTCGTCGG
CTTCACCGCCGCCTGGCAGCTCGCGCTCGTCACGCTCGCCGTCGTACCGCTCATCGCCGTCATCGGGGGGC
TCAGCGCCGCCGCGCTCTCCAAGCTCTCCGCCAGGAGCCAGGACGCGCTCTCCGGCGCCAGCGCCATCGCC
GAGCAGGCGCTGGCGCAGATACGGATCGTGCAGGCCTTCGTCGGCGAGGAGCGCGCCATGCGGGCGTACTC
GGCGGCGCTGGCCGTCGCGCAGAAGATCGGCTACCGGAGCGGCGTCGCCAAGGGGCTCGGGCTCGGCGGCA
CCTACTTCACCGTCTTCTGCTGCT
```
