# Supplementary material for: Fine-Mapping and Identification of a Candidate Gene Underlying the d2 Dwarfing Phenotype in Pearl Millet, Cenchrus americanus (L.) Morrone
Source: G3 (Bethesda). 2013 Mar 1;3(3):563–72. doi: 10.1534/g3.113.005587 (PMC3583462; doi:10.1534/g3.113.005587)
Supplement: Supporting Information [file supp_3.3.563_FileS2.pdf]

File S2

Sequence of DNA fragments obtained with primer set Ca_Sb07g023730F10/R10 in the tall inbred line ICMP 451 (A) and in the dwarf inbred line Tift 23DB (B). The synonymous SNP that differentiates the ICMP 451 and Tift 23DB sequences is shown in red.

```
A. ICMP 451_Ca_Sb07g023730F10/R10
GCAGGTTCTCCTTGATGCTCGTCGCGAACAGCGTCGGCTCCTGACTCACCAGGCCCATCTGCTGCCGGAGC
CACCGGAGCTTCAGGCTCTTGAGGTCATGCCCGTCCAGCAGGATCTCACCTGTTGCAATTGCAATTCCCAT
CGCTCATCAGAATAGTAGTTTGTTTTCTAGTAGTAGACTTTTCTGCCTATTACTAGAGGATGCCTATGAAT
GACTGTGACTGGACAAGAGGATGATGCAGCGCAAAGGAAGCAGCCCATGAGGCCGGCAAGCTCTGCTCCAA
TCCAAGCACTACTTGCTTGGTTGCTTGCACCACCCCCACCCAGCAATGGCGGGCAATGCTGATTACTGATG
GCTCCAATCCAACAAGAGAATGTAACCAACAACGGGTATACCTGCGCTGGGGTCGTAGAACCTCTCGATGA
GCGACACGACGGTGCTCTTCCCGGAGCCGGAGCTGCCGACCAGCGCGATGGTCTTCCCGGCGGGCACGGTG
AGGGAGAAGGCGCGCAGGATGGGCACGTCCGGCCGCGTCGGGTACGCGAAGTCGACGCCCCGCATCTCTAC
CCGCCCCGTGACGGACTCCAGCTCCACCTGGCCGTCCCGCGAGATCCCCGGCTTGTGGTCGATGATGCGGA
AGATCTTGGCCGCCGCGACGCGCGCCTTGGCGAAGGCGGCCATGCTCGGCGCCGACTGCCCCAGCGCGATG
CCCCCGATCATGACGGAGAACATGGTGGCGATGGCGAGCCCCCCGTTGGTGCGGTGGCCGCGCACCAGGTG
GCCCCCGTACCAGAGCAGCAGCCCGTAGCAGCAGAAGACGGTGAAGTAGGTGCCTCCGAG

B. Tift 23DB_Ca_Sb07g023730F10/R10
GCAGGTTCTCCTTGATGCTCGTCGCGAACAGCGTCGGCTCCTGACTCACCAGGCCCATCTGCTGCCGGAGC
CACCGGAGCTTCAGGCTCTTGAGGTCATGCCCGTCCAGCAGGATCTCACCTGTTGCAATTGCAATTCCCAT
CGCTCATCAGAATAGTAGTTTGTTTTCTAGTAGTAGACTTTTCTGCCTATTACTAGAGGATGCCTATGAAT
GACTGTGACTGGACAAGAGGATGATGCAGCGCAAAGGAAGCAGCCCATGAGGCCGGCAAGCTCTGCTCCAA
TCCAAGCACTACTTGCTTGGTTGCTTGCACCACCCCCACCCAGCAATGGCGGGCAATGCTGATTACTGATG
GCTCCAATCCAACAAGAGAATGTAACCAACAACGGGTATACCTGCGCTGGGGTCGTAGAACCTCTCGATGA
GCGACACGACGGTGCTCTTCCCGGAGCCGGAGCTGCCGACCAGCGCGATGGTCTTCCCGGCGGGCACGGTG
AGGGAGAAGGCGCGCAGGATGGGCACGTCCGGCCGCGTCGGGTACGCGAAGTCGACGCCCCGCATCTCTAC
CCGCCCCGTGACGGACTCCAGCTCCACCTGGCCGTCCCGCGAGATCCCCGGCTTGTGATCGATGATGCGGA
AGATCTTGGCCGCCGCGACGCGCGCCTTGGCGAAGGCGGCCATGCTCGGCGCCGACTGCCCCAGCGCGATG
CCCCCGATCATGACGGAGAACATGGTGGCGATGGCGAGCCCCCCGTTGGTGCGGTGGCCGCGCACCAGGTG
GCCCCCGTACCAGAGCAGCAGCCCGTAGCAGCAGAAGACGGTGAAGTAGGTGCCTCCGAG
```
