# Supplementary material for: Fine-Mapping and Identification of a Candidate Gene Underlying the d2 Dwarfing Phenotype in Pearl Millet, Cenchrus americanus (L.) Morrone
Source: G3 (Bethesda). 2013 Mar 1;3(3):563–72. doi: 10.1534/g3.113.005587 (PMC3583462; doi:10.1534/g3.113.005587)
Supplement: Supporting Information [file supp_3.3.563_TableS1.pdf]

Table S1 Primer sequences, their melting temperature and location in the sorghum, rice and foxtail millet genomes of markers mapped in the *d2* region

Marker		Primer sequences	Tm (^{0}C)	Corresponding sorghum gene	Location in sorghum genome (Mb)[1]	Corresponding rice gene	Location in rice genome (Mb)[1]	Corresponding foxtail millet gene	Location in foxtail millet genome (Mb)[1]
B224C4P2	F2	TTCCTGGGATCAAAAGGTTC	59-52	-	-	-	-	-	-
	R1	CCATATCTCCAAGCTGCATAG							
Ca_Sb07g024020	F	CCACGCGCTTCTTCCTCC	61	Sb07g024020	7: 59.04	Os08g0560300	8: 28.15	Si015992m	VI: 35.69
	R	TCCTCCACCTCGGACACG							
Ca_Sb07g023910	F	CATGCACTGACGAGGTTGG	53	Sb07g023910	7: 58.93	Os080562100	8: 28.23	Si013632m	VI: 35.76
	R	TGCCCCATTTTTGGATGAGC							
RGR1963	2F	GTACGCCTACAGTCAAGGTG	59-52	Sb07g023850	7: 58.82	Os08g0562700	8: 28.27	Si013315m	VI: 35.80
	2R	CCAAGCTCGTGTTGCGCAG							
Ca_Sb07g023840	F	ACATCCTCCCCTCCGTATTC	53	Sb07g023840	7: 58.81	Os08g0562800	8: 28.27	Si013571m	VI: 35.81
	R	ATGGAGAAGCTGTCCCTGAAG							
Ca_Sb07g023810	F	CTTGGCTCACCATGTGGTG	61	Sb07g023810	7: 58.78	Os08g0563400	8: 28.30	Si015156m	VI: 35.83
	R	GACTCTAGTTCTGCTTGCAG							

Ca_Sb07g023730	F1	TACGCCTTCTACTTCCTCGTC	61	Sb07g023730	7: 58.61	Os080564300	8: 28.36	Si013123m	VI: 35.86
	R5	AGCAGCAGAAGACGGTGAAGTAG							
Ca_Sb07g023630	F	GCTCTTTTCAGCAACCCCTTC	53	Sb07g023630	7: 58.53	Os08g0564800	8: 28.39	Si013125m	VI: 35.88
	R	CGCTAACTGCAGCAGCAAGG							
Ca_Sb07g023520	F	GCCATTTTGGCGGAGATTGC	53	Sb07g023520	7: 58.42	Os080566000	8: 28.44	Si013435m	VI: 35.92
	R	CTGCGCACCAGCAATAGG							
Ca_Sb07g023500	F	GCTTTAGACGGTGCAGAC	59-52	Sb07g023500	7: 58.40	Os08g0566400	8: 28.46	Si015293m	VI: 35.94
	R	GAAGCTCATCCAATCCCTC							
Ca_Sb07g023470	F	AGCTTCAGCTCCGTGCTTC	59-52	Sb07g023470	7: 58.39	Os080566600	8: 28.47	Si015371m	VI: 35.95
	R	GGAATCCGAGCTTCTCTCC							
Ca_Sb07g023460	F2	GCATCTGTCCAACTGACAGC	59-52	Sb07g023460	7: 58.38	Os080566900	8: 28.48	Si014505m	VI: 35.95
	R2	ATCTTGCTGCCTTGAGGTTC							
Ca_Sb07g023440	F	CGCCTTCGACATGGACGAC	53	Sb07g023440	7: 58.37	Os080567100	8: 28.49	Si015189m	VI: 35.96
	R	CTTGTCGTGGCGGTAGTCG							
Ca_Sb07g023430	F	TGATAAACCCAGCAACCTTGG	53	Sb07g023430	7: 58.37	Os12g0641500	12: 27.75	Si013129m	VI: 35.97
	R	GAATCAAGTACACAGCTATGC							

PSMP344	F	CGTCCTGTACCTCAGCCTTG	59-52	Sb07g007800	7: 12.59-12.61	-	-	Si015622	VI: 9.10
	R	ATGCTACACCTGTCAGAACTAAGAG							
PSMP344	F2	GGCTTGTCACGTCCTGTACC	53	Sb07g007800	7: 12.59	-	-	Si015622	VI: 9.10
	R2	TCAATCATGCAGCAGCAATC							
PSMP305	F	TGCAATAGTTTCCAGTTGTC	53	-	-	-	-	-	VI: 1.36
	R	TCAACAATTTGGTACAGACG							

[1]The number before the colon indicates the chromosome number; the number following the colon is the chromosomal location in Mb.
